# Older People in the Platform Economy

**DOI:** 10.3389/fsoc.2019.00008

**Published:** 2019-02-18

**Authors:** Eva Berde

**Affiliations:** ^1^Department of Microeconomics, Corvinus University of Budapest, Budapest, Hungary; ^2^Demography and Economics Research Centre, Corvinus School of Economics, Corvinus University of Budapest, Budapest, Hungary

**Keywords:** older workers, platform, online survey, Bayesian game, age discrimination

## Abstract

Since the platform economies have emerged, there are many new ways how people can perform earning activities. These new opportunities concern older people too. However, some authors write that the actors of the platform economy are mainly younger people and it is not typical that older people work through platforms. We cite the opposite case of Oszkar (Hungarian On-line Passenger Intermediary System) where the ratio of older (55+) drivers to the total number of chauffeurs increased in time. At the same time, 30–40% of all drivers do not declare their age. In our paper, we analyse why older people do not declare their age. Using the results of our online survey and a Bayesian game theoretic model we prove that in the equilibrium state older people are frequently against letting others know their real age when they apply to work through a platform. We think that the cause is still existing age discrimination

## Introduction

Increased longevity, and at the same time, greater healthy life expectancy makes it possible to work for a longer time as well. However, in the majority of European welfare states up until the mid-1980s older generations could enjoy the comfort and relative material well-being of retirement at a comparatively early age. It was generally accepted that it was the “duty” of older people to retire and make room in the workplace for young people.

An extreme example is the “Walmart effect,” described by Ilmakunnas and Maliranta (2007), whereby management sought to replace even the middle-aged by more efficient younger workforce. Starting from the end of the 1980s retirement age in most European countries began to rise, and governments tried to popularize the value of older people's labor. PwC (2018) using their famous Golden Age Index—which calculates the percentage of 55–64 year old active in the labor market—estimated that in the year 2017 the Gross Domestic Product (GDP) of the Organization for Economic Co-operation and Development (OECD) countries would have been higher by $3.5 trillion if they had managed to keep all older adults on the labor market until they reached retirement age. The government's intent, however, is frustrated not only by older people's lack of ambition but also by beliefs ingrained in society, as well as corporate level strategies.

In our paper, we examine a particular manifestation of societal beliefs. Our first hypothesis states that older people suffer—or at least they feel that they suffer—negative discrimination not only when applying for traditional jobs, but also when they seek work using online platforms. Our second hypothesis states that older people adapt to these conditions and either misrepresent or do not declare their age.

The beginning of the whole story was when we saw the data analysis from the Hungarian carpooling platform Oszkar^1^ in Berde and Tokes (2018), which shows that on average 30–40% of the chauffeurs using the service do not enter their age on their profile. Of course, this does not mean that only older people omit their age. It is likely that others, especially the very young, do the same. Also, Berde and Tokes (2018) show that contrary to the claim of Huws et al. (2017) older adults do like to use online platforms to look for work. At least in the case of Oszkar, the ratio of drivers aged 55 years or more has increased while the total number of drivers was increasing as well. Thus, how older people behave while they seek work on online platforms may be a question of particular importance.

We prove our hypothesis, concerning the omission of age on profiles, by way of an online survey. The description of the survey is in the second part of our article, where we present its results as well. In the third part, we use a well-known game-theoretic model to support further our argument that older adults may feel the need not to disclose their age. Finally, we conclude our findings.

## The Online Survey

Our online survey consists of five questions. The full text of the survey can be found in the Appendix. Respondents were first asked their year of birth and gender. Then we inquired if they believed when someone was thinking about using the services they offered through an online platform (e.g., chauffeuring services, tutoring, cleaning) the prospective clients would take their age into consideration. The next question asked whether respondents would enter their age on the platform if it was possible, but not mandatory. Lastly, if they chose to enter age, we asked what it would be.

We propagated the online survey on special mailing lists and Facebook groups where most participants were more than 50 years old, sometimes significantly older. Links to the survey were distributed in June 2018 and responses were collected until the end of August 2018. We got 140 responses. The youngest responder was 24-year-old, the oldest 78-year-old. For the sake of uniformity, we removed those under the age of 50 from the sample, thereby retaining 131 responses for evaluation. The distribution of the answers in the remaining sample is shown in Table 1 below.

**Table 1:** Distribution of answers in the 131 responses submitted by people over the age of 50.

**Gender**	**Female: 59.54%**	**Male: 40.46%**
Displayed age would affect the number of clients who order your service	Yes: 80.92%	No: 19.08%
Declare true age on profile	Declare: 54.96%	Do not declare: 45.04%
Among those who do not think age matters with respect to the number of clients, declare true age on the profile	Declare: 100%	
Among those who think age matters with respect to the number of clients, declare true age on the profile	Declare: 44.34%	Do not declare: 55.66%

From the figures of Table 1, it is important to highlight that in our sample 45% of people older than 50 would not voluntarily declare their true age on their profile. It is also interesting to note that 19% of respondents think their age would not matter when someone is considering engaging their services. All such respondents would declare their real age on their profile.

Only five of the respondents said that they would declare an age lower than their true age—albeit only by a few years—in order to seem younger. They represent merely 3.82% of the sample. Therefore, we decided not to put these people into a separate category for our analysis, but instead group them with the people who declared their real age. Using the survey results we rejected our previous belief that older adults will try to misrepresent their age when offering services^2^.

Our online survey cannot be considered representative since it was disseminated in a particular way. Belonging to an online mailing list or a Facebook group constitutes self-screening. Our survey does show however that older adults do think age is a significant factor in whether their offered services are engaged or not. In the next part of our study, we use the results of our survey to show that in a Bayesian game such as the one presented by Cho and Kreps (1987) and Kreps (1990) older adults may similarly attempt to hide their age.

## The Model

### The Background of Our Model

Our model uses two theoretical game concepts, both classic by present day standards. The first one is job market signaling, the second is the transformation of games of incomplete information into games of imperfect information. The concept of job market signaling was first introduced by Spence (1978). In this concept, the employer offers a contract, wherein the wage paid is conditional on the level of education the employee can certify. However, the employee's level of competence is only known to them, and is not necessarily reflected by their qualifications. A less competent person with a college degree may garner a higher salary than a similarly competent person without a college degree. Though signaling was applied to the job market directly in Spence (1978), similar ideas are formulated in Arrow (1973), Mincer (1974), and Stiglitz (1975). Since the seminal work of Spence, signaling is one of the most frequently used tools when studying employee contracts. Some frequently cited applications are Lazear and Rosen (1981), Hanushek (1986), Heckman et al. (2006), and Slee (2017). Of these, Slee (2017) offers the conclusions most relevant to our subject. Slee points out that the rating systems of gig economy jobs performed through online platforms make it seem as though the people performing the job have been rated objectively, based on the quality of their work. However, in real life these ratings may contain even more prejudice than conventional ratings given by supervisors. Workers in the gig economy are compelled to send signals that counter this prejudice, so they get better ratings (e.g., they may try to hide the fact that they belong to a local ethnic minority).

Information sets can be used in situations when some players are aware of a specific piece of information but others are not. This piece of information is sometimes referred to as the type of player who is aware of it. This may be their age, level of competence, etc. Harsanyi (1967; 1968a; 1968b) has shown how to transform games of incomplete information into games of imperfect information, by introducing “Chance” as the first mover. “Chance” has no payoffs, but it determines the types of the players. Players may signal their type via actions. e.g., in the Spence (1978) model the more competent people may complete their university education with less effort, and are therefore more likely to get a degree. However, a player may try to signal a type different from their own, and this may increase their payoff, as with the less competent person with a degree in the previous example. The other player can only form a belief about the type based on the signal received, so all types with the same signal fall into the same information set. The job market is modeled using this technique in Roth and Sotomayor (1992). Osborne and Rubinstein (1994) also contains applications of the Harsanyi transformation, where in addition they emphasize the role of outside circumstances. They also say that the circumstances depend on the behavior of the players, and conversely the behavior of the players depends on the circumstances too. Our own model, introduced below, is built upon the same principles.

### The Structure of the Model and Its Results

In our model, there are two types of older adults' chauffeurs for hire. The first type thinks his age does not matter; the clients will engage his services regardless of his age. We denote this type by R, short for “Regardless.” The second type believes that age matters to the clients and older chauffeurs get fewer engagements. We denote this type by M, short for “Matters.” In order to offer their services, both types register on an online platform, where they fill out a data sheet. It is not required to answer all questions. Specifically, it is not mandatory to declare their age. In reality, this is not verified by the platform, hence misrepresentation is also possible, but in our model, we do not consider this possibility. The only options are declaring their real age or not declaring any age at all.

Of course, passengers are more likely to meet young chauffeurs than old chauffeurs, but we restrict ourselves to this latter case. When the potential passenger encounters a posting by the chauffeur, they either engage their services, or they do not. One piece of information used by the passenger to make this decision is the age of the chauffeur. However, this may be missing. In this case, the passenger does not know why the chauffeur did not declare their age; it may be because they are too old, or they may have simply glossed over it when they filled out the data sheet.

The payoffs are similar to the ones in the game presented by Cho and Kreps (1987). Passengers receive a payoff of 1 if they interact with the old chauffeur according to their type. In our model, this means engaging the services of those who think they will be hired regardless of their age, and not engaging the services of those who think their age matters. If passengers choose the wrong action, they receive a payoff of 0. We summarize passengers' payoffs below:

Engage services of type *R*: 1.
Do not engage services of type R: 0.
Engage services of type M: 0.
Do not engage services of type M: 1.

However, the passenger cannot directly observe the type of the chauffeur. They can only observe if they have declared their age or if they have not. Declaring their age is a signal sent by the chauffeur, which yields a type dependent direct payoff, and it may influence the passenger's decision as well. We summarize the direct payoffs of drivers from their signal: Type *R* and declares age: 1.

Type *M* and declares age: 0.
Type *R* and does not declare age: 0.
Type *M* and does not declare age: 1.

The chauffeurs' payoff depends more on whether or not the passengers engage their services. The chauffeurs' payoffs resulting from the passenger's decision are given below:

Services are engaged:
Services are not engaged: 0.

Based on the previous payoffs, the payoff of the type *R* chauffeurs, if they declare their age and their services are engaged by the passenger, is 3. If they declare their age, but their services are not engaged, their payoff is 1. If they do not declare their age, but their services are engaged, they receive a payoff of 2. If they do not declare their age and their services are not engaged their payoff is 0. The payoff of the type *M* chauffeur is similarly addictive. If type *M* drivers declare their age and their services are engaged, they get a payoff of 2. However, if they do not declare their age and their services are not engaged, they get a lower payoff, 1. Therefore, if declaring their age can move the passenger to engage their services, it is worthwhile to do so, even though this goes against the character of the type *M* chauffeur.

Now we proceed to determine the equilibrium of our Bayesian game based on Cho and Kreps (1987) using the reasoning presented in Binmore (1992). The starting player is Chance, who decides the type of the chauffeur with exogenously given probabilities. Based on the results of our survey, in our game, the probability of type *R* will be 19.8%. The game has no pure strategy equilibrium [For a detailed proof see Binmore (1992) page 464, figure 10.11. In that example, the probabilities differ from ours, but the same calculations prove that in our game there is no pure strategy equilibrium either]. Since we are dealing with a game of incomplete information, we will use the Harsanyi transformation to make it into a game of imperfect information and solve that. This is why we introduce Chance as the starting player. We denote the probability that type *R* drivers do not declare their age by *NG* (None Given), and we denote the probability that they declare their age by *D* (Declare). Of course, NG+D = 1. In the case of type *M*, the same probabilities are denoted by *ng* and *d*, respectively.

The passenger, as we have already stated, can only observe the signal sent by the chauffeur, that is whether or not the chauffeur has declared their age. Both in the “Declare” and the “None Given” information sets the passengers will only engage the chauffeur's services if they believe the chauffeur has a higher probability of being type *R* than of being type *M*.

In the “None Given” information set services are engaged i

(1) $$\begin{array}{l} \frac{0.198NG}{0.198NG+0.802ng}>\frac{0.802ng}{0.198NG+0.802ng} \end{array}$$

that is if *NG* > 4.05*ng*.

(2) $$\begin{array}{l} \text{Services are not engaged if NG}<4.05\text{ng} \end{array}$$

In order for the passenger to randomize we must have

(3) $$\begin{array}{l} NG=4.05ng \end{array}$$

Therefore

(4) $$\begin{array}{l} 1-D=4.05-4.05d \end{array}$$

which yields

(5) $$\begin{array}{l} 4.05d=3.05+D \end{array}$$

In the “Declare” information set services are engaged if

(6) $$\begin{array}{l} \frac{0.198D}{0.198D+0.802d}>\frac{0.802d}{0.198D+0.802d} \end{array}$$

that is if

(7) $$\begin{array}{l} D>4.05d \end{array}$$

Services are not engaged if

(8) $$\begin{array}{l} D<4.05d. \end{array}$$

Based on (5, 8) if the passenger randomizes in the “None Given” information set, they will not engage services in the “Declare” information set. Thus, type *M* will not declare their age, as that would mean *ng* = 1, and together with (3) it would imply *NG* = 4.05, which is impossible.

This means that the only mixed equilibrium is such that the passenger randomizes in the “Declare” information set.

(9) $$\begin{array}{l} D=4.05d \end{array}$$

This means that

(10) $$\begin{array}{l} 1-NG=4.05-4.05ng \end{array}$$

(11) $$\begin{array}{l} NG=4.05ng-3.05 \end{array}$$

(12) $$\begin{array}{l} NG<4.05ng \end{array}$$

thus services are not engaged in the “None Given” information set. Therefore, type *R* will always declare their age, so *D* = 1 and based on (9) *d* = 0.2469. This shows that type *M* will randomize between the “None given” and “Declare” behavioral strategies. In case of “None given,” type *M* receives a payoff of 1, and his expected payoff must be the same when he chooses “Declare.” Let us denote the probability that services are engaged in the “None given” information set by θ. Type *M* will randomize if

(13) $$\begin{array}{l} 1=0+2\theta \end{array}$$

which yields

(14) $$\begin{array}{l} \theta =\frac{1}{2} \end{array}$$

Thus, type *R* chauffeurs will always declare their age, while type *M* chauffeurs will declare their age with probability 0.2469. In the “None Given” information set the passengers will never engage the chauffeur's services, while in the “Declare” information set they will do so with probability 0.5.

According to our survey, less than half of the *M* type chauffeurs would declare their age. Our game theoretical model based on Cho and Kreps (1987) yields an even lower ratio, although this clearly depends on the exact numerical values in the payoff function. The order of these values is essential: If a type *M* chauffeur does not declare their age, they get a lower payoff than if they do declare their age and as a result, their services are engaged. This is in accordance with the results of our survey even though 81% of the respondents thought that age matters, 44% of them would still declare their age despite their misgivings.

## Conclusions

Due to the small number of observations and to the self-screening in the online groups used to propagate our questions, the survey cannot be considered representative, but it does provide clear guidance about whether people think age matters. The high ratio of affirmative answers allows us to conclude that most old people feel that there is age discrimination in the labor market of the platforms. This result is further supported by our model based on Cho and Kreps (1987). We also demonstrated both by the survey and the model, that people recognize society's attitude toward older adults, and in order to be successful in the platform, they adjust their behavior accordingly.

All this confirms that the situation is similar to the one described in Osborne and Rubinstein (1994). The behavior of older chauffeurs is affected by their environment. Those who think their age matters when considering to offer their services frequently do not reveal their age. These chauffeurs probably formed their beliefs after previous negative experiences. However, by omitting their birth year they decrease the ratio of older chauffeurs who have admitted their real age. This may reaffirm the public's belief that older chauffeurs are less capable drivers, and hence their services should not be engaged.

This creates a vicious cycle. It is very difficult to change underlying attitudes. Our results call attention to the fact that in order to lengthen labor market participation mere governmental intent will not suffice. To be able to retain older adults longer a shift in societal attitude is required as well.

## Ethics Statement

An ethics approval was not required for this study as per Corvinus University of Budapest (Hungary) guidelines, and national regulations and the informed consent of the participants was implied through survey completion.

## Author Contributions

EB was the only author of the article. She managed the on-line survey and elaborated its results. She described the game theoretic model using the cited literature as well.

### Conflict of Interest Statement

The author declares that the research was conducted in the absence of any commercial or financial relationships that could be construed as a potential conflict of interest.

## Footnotes

^1^Upon its founding in 2007 Oszkar was basically a carpooling service. Based on Oszkar.hu's present day data Berde and Tokes (2018) have concluded that commercial postings have become an ever-increasing majority.

^2^We did not consider how likely it is that someone who would lie would also admit that they would lie. Maybe they wish to hide their mistrustful behavior.

## Appendix

Questions of the online survey (translated from Hungarian)

1. What is your gender?
  - Female
  - Male
2. Year of birth
3. The image that you are advertising service through an online platform. E.g., tutoring, chauffeuring services, cleaning, etc. When you register on the platform you may enter your age, but this is not verified by anyone so that you can omit this declaration. Do you think your age will affect the number of your clients?
  - It will
  - It will not
4. Would you enter (any) year of birth?
  - I would probably not.
  - I probably would
5. What year of birth would you enter? In case you answered, “I would probably not.” in the previous question, write zero (0) now. Don't forget; you are not obliged to enter your genuine year of birth. ……
